# CC Chemokine Ligand 18 Correlates with Malignant Progression of Prostate Cancer

**DOI:** 10.1155/2014/230183

**Published:** 2014-08-17

**Authors:** Guo Chen, Yu-xiang Liang, Jian-guo Zhu, Xin Fu, Yan-fei Chen, Ru-jun Mo, Liang Zhou, Hao Fu, Xue-cheng Bi, Hui-chan He, Sheng-bang Yang, Yong-ding Wu, Fu-neng Jiang, Wei-de Zhong

**Affiliations:** ^1^Department of Urology, Guangdong Key Laboratory of Clinical Molecular Medicine and Diagnostics, Guangzhou First People's Hospital, Guangzhou Medical University, Guangzhou 510180, China; ^2^Department of Urology, Guizhou Provincial People's Hospital, Guizhou 550002, China; ^3^Department of Urology, Nanhua Hospital Affiliated Nanhua University, Hengyang, Hunan 421002, China; ^4^Department of Urology, Guangdong General Hospital, Guangzhou 510080, China; ^5^Guangdong Provincial Institute of Nephrology, Southern Medical University, Guangzhou 510515, China

## Abstract

*Background and Aim*. CC chemokine ligand 18 (CCL18) promotes malignant behaviors of various human cancer types. However, its involvement in human prostate cancer has not been fully elucidated. The aim of this study was to investigate the role of CCL18 in PCa. *Methods*. Expression of CCL18 at mRNA and protein levels was detected using real-time qRT-PCR and immunohistochemistry analysis. We analyzed the associations of CCL18 expression with clinical features of human PCa. The effects of PCa cell migration, invasion, and apoptosis were tested. The efficiency of CCL18 on prostate tumor growth was assessed in a subcutaneous xenograft model. *Results*. CCL18 expression was upregulated (both *P* < 0.01) in PCa tissues compared with those in noncancerous prostate tissues. CCL18 upregulation was correlated with high Gleason score (*P* = 0.034) of patients with PCa. rCCL18 stimulation in PCa cells promoted cell migration and invasion but decreased DU145 cells apoptosis rate. Furthermore, subcutaneous homografts models showed the increased tumor growth and tumor vascularization with the CCL18 stimulation, and the expression of Ki67, PCNA, and CD31 in CCL18 stimulation mice was also significantly increased. *Conclusions*. Our data offer the convincing evidence that the upregulation of CCL18 may be involved in the malignant progression of PCa.

## 1. Introduction

Prostate cancer (PCa) represents the most commonly solid cancer and the second leading cause of cancer-related deaths among men in the United States [1]. It is a clinically heterogeneous-multifocal disease and the incidence is continuously rising. Carcinogenesis of PCa is a multistep process, involving both genetic insults to epithelial cells and changes in epithelial-stromal interactions [2]. Most PCa patients progress to the androgen-independent stage, and although androgen ablation therapy, surgery, and radiation therapy are effective for the treatment of local PCa, no effective treatments are available [3]. In spite of several clinical parameters, such as serum prostate specific antigen (PSA) levels, age and underlying health of men, the extent of tumor spread, appearance under the microscope, and the response to initial treatment, which may provide some prognostic utility in the treatment settings, there are currently no definitive clinical methods that can reliably predict the responses to clinical therapies for PCa [4]. Therefore, it is of great significance to identify novel and effective biomarkers involved in the proliferation, migration, and invasion of PCa cells in order to strengthen the efficiency of early diagnosis and to help create the efficient therapeutic strategies of this type of cancer.

Chemokines, a family of chemotactic cytokines, act through seven-transmembrane domain G protein-coupled receptors on their target cells and play a role in various biological and pathological processes, such as migration of leukocytes, angiogenesis, and tumor growth [5]. Among chemokines, the CCL chemokines, the largest family of chemokines, are able to attract monocytes, macrophages, T cells, B cells, eosinophils, dendritic cells, mast cells, and natural killer cells [6]. As a novel defined member of CC chemokines, CC chemokine ligand 18 (CCL18) was originally discovered as pulmonary and activation-regulated chemokine (PARC), dendritic cell- (DC-) chemokine 1 (DC-CK1), alternative macrophage activation-associated CC chemokine-1 (AMAC-1), and macrophage inflammatory protein-4 (MIP-4) [7]. It is predominantly produced by monocyte-derived cells with M2 phenotype [8]. CCL18 contains an open-reading frame encoding a polypeptide of 89 amino acids. Excessive production of CCL18 in M2-macrophages was demonstrated in various chronic inflammations and fibrotic diseases [9]. Besides these, CCL18 has been demonstrated to promote the invasiveness of cancer cells by triggering integrin clustering and enhancing their adherence to the extracellular matrix (ECM) [10]. It is conceivable that CCL18 can act in a paracrine fashion during tumor initiation/establishment. In gastric cancer, CCL18 overexpression has been reported to be associated with a better survival in patients [11]. In opposition to gastric cancer, CCL18 could promote the tumor malignant progression of breast cancer [10], ovarian cancer [12], bladder cancer [13], pancreatic cancer [14], and skin diseases [15], and its upregulation could predict poor prognosis in these cancers. These findings suggest that the involvement of CCL18 in different human cancers may depend on various cancer types.

However, perplexing involvement of CCL18 in PCa has not been fully elucidated. Thus, the aim of the current study is to investigate the expression patterns, the clinical significance, and the oncogenic roles of CCL18 in PCa.

## 2. Materials and Methods

### 2.1. Patients and Tissue Samples

The study was approved by the Research Ethics Committee of Guangzhou First People's Hospital, Guangzhou Medical University, China. Informed consent was obtained from all of the patients. All specimens were handled and made anonymous according to the ethical and legal standards.

For quantitative real-time reverse transcriptase PCR (qRT-PCR) analysis, frozen samples of primary PCa tissues and adjacent benign prostate tissues were collected from the tissue bank at Guangzhou First People's Hospital. For immunohistochemistry analysis, (TMA) including 80 primary PCa tissues and 96 adjacent noncancerous prostate tissues for CD68 staining, 80 primary PCa tissues and 95 adjacent noncancerous prostate tissues for CCL18 staining were obtained from Shanghai Outdo Biotech Co, Ltd. (Cat number: HPro-Ade180PG-01), including the detailed clinical information. The detailed information on the clinical features of all patients in this study is shown in Table 1.

### 2.2. Cell Culture

Human prostate carcinoma cell lines, LNCaP and DU145, and murine PCa RM-1 cells were purchased from the American Type Culture Collection (Manassas, VA, USA) and were cultured in RPMI 1640 medium (Hyclone, USA) supplemented with 10% fetal bovine serum (Gibico, USA), 2 mM L-glutamine, and antibiotics. All cell lines were maintained at 37°C in a humidified chamber supplemented with 5% CO_2_.

### 2.3. Tumor Homografts in Mice and Treatment

All animal experiments were approved by the Ethics Committee of Guangzhou First People's Hospital, Guangzhou Medical University, China, and conducted accordingly.

For homotransplantation, 30 male C57BL/6 mice aged 6~8 weeks were purchased from Guangdong Medical Lab Animal Center. Recombinant CCL18 (rCCL18) was obtained from PeproTech (Cat: 300-34.USA). Thirty mice were divided into two groups: control group (mice were inoculated subcutaneously in the backs with 1 × 10^5^ RM-1 cells in 0.2 mL of physiological saline, *n* = 10) and rCCL18 stimulation group (mice were inoculated subcutaneously in the backs with 1 × 10^5^ RM-1 cells in 0.2 mL of physiological saline with 20 ng/g rCCL18, *n* = 20). After homotransplantation, treatment commenced by every 3 days subcutaneous injection of rCCL18 at 1 ng/g or equivalent amounts of solvent for additional 28 days for rCCL18 stimulation group and control group, respectively. The tumor size was measured every 7 days with a caliper; the tumor volume was calculated according to the formula 0.5 × *L* × *W* ^2^ (*L* = length; *W* = width).

### 2.4. Real-Time Quantitative Reverse Transcriptase-PCR

Quantitative PCR was used to examine the expression status of CCL18 mRNA in PCa and noncancerous prostate tissues. The cDNA templates for qRT-PCR were synthesized from RNA samples. The primers of CCL18 genes and *β*-actin which was used as an internal reference were shown as follows: the sequence of CCL18 forward primer is 5′-CTC TGC TGC CTC GTC TAT ACC T-3′; the reverse primer is 5′-CTT GGT TAG GAG GAT GAC ACC T-3′. The sequence of *β*-actin, forward primer is 5′-AGC GAG CAT CCC CCA AAG TT-3′; the reverse primer is 5′-GGG CAC GAA GGC TCA TCA TT-3′. CCL18 primers are intron-spanning. Gene expression was determined using SYBR Green PCR mix (Toyobo, Japan) and 0.2 *μ*g of template. Real-time PCR was performed on a MyiQ.2 Two-Color Real-Time PCR Detection System (Bio-Rad), using the following amplification conditions: 5 min, 95°C followed by 40 cycles of 10 seconds 95°C, 20 seconds 60°C, and 20 seconds 72°C. All assays were carried out in triplicate. CT values were determined using the IQ5 software (Bio-Rad). Gene expression in each sample was normalized with *β*-actin expression. Relative quantification of target gene expression was evaluated using the comparative cycle threshold (CT) method.

### 2.5. Immunohistochemistry Analysis

The specimens were fixed in 10% neutral buffered formalin and subsequently embedded in paraffin. The paraffin-embedded tissues were cut at 4 *μ*m and then deparaffinized with xylene and rehydrated for further H&E or peroxidase (DAB) immunohistochemistry staining employing DAKO EnVision System (Dako Diagnostics, Switzerland). Briefly, following a brief proteolytic digestion and a peroxidase blocking of tissue slides, the slides were incubated overnight with the primary antibodies against CCL18 (Abcam, Cat: ab104867, at a dilution of 1 : 800), Ki67 (Affinit, Cat: AF0198, at a dilution of 1 : 100), PCNA (Boster, Cat: BM0104, at a dilution of 1 : 800), and CD31 (ZSGB-BIO, Cat: ZA-0568, at a dilution of 1 : 200) at 4°C, respectively. After washing, peroxidase labeled polymer and substrate chromogen were then employed in order to visualize the staining of the interested protein. In each immunohistochemistry run, negative controls were stained with isotype-matched control IgG.

Following a hematoxylin counterstaining, immunostaining was scored by two independent experienced pathologists, who were blinded to the clinicopathological data and clinical outcomes of the patients. The scores of the two pathologists were compared and any discrepant scores were trained through reexamining the staining by both pathologists to achieve a consensus score. The number of positive-staining cells in ten representative microscopic fields was counted and the percentage of positive cells was calculated. Given the homogenicity of the staining of the target proteins, tumor specimens were scored in a semiquantitative manner. The percentage scoring of immunoreactive tumor cells was as follows: 0 (0–5%), 1 (6–25%), 2 (26–50%), 3 (51–75%), and 4 (>75%). The staining intensity was visually scored and stratified as follows: 0 (negative), 1 (weak), 2 (moderate), and 3 (strong). A final immunoreactivity score (IRS) was obtained for each case by adding the percentage and the intensity score.

### 2.6. Cell Invasion Assay

The transwell inserts (8 *μ*m pores) were filled with 50 *μ*L of a mixture of serum-free RPMI1640 medium and matrigel (1 : 10; BD Biosciences, USA). The inserts were then placed in 24-well tissue culture plates (Transwell, Corning, USA) containing 10% FBS-medium. After solidification by incubation in 37°C for 4 hour, 5 × 10^4^ cells in 200 *μ*L medium were placed in upper chambers. Then, 500 *μ*L serum-free RPMI1640 medium with or without rCCL18 was placed in lower chambers. Following 24 hours of incubation at 37°C with 5% CO_2_ and in culture medium with mitomycin to stop the mitosis, the membranes were fixed with 10% formalin and stained with 0.05% crystal violet. The number of cells that migrated through the pores was assessed and the data were expressed as of three independent experiments.

### 2.7. Cell Migration Assay

For the scratch wound-healing motility assay, a scratch was made with a pipette tip when the cells reach the confluence. After being cultured under standard conditions with mitomycin for 48 hours, plates were washed twice with fresh medium to remove nonadherent cells and then photographed. The cells that migrated from the wound edge were counted and the data were expressed as of three independent experiments.

### 2.8. Apoptosis Assay

Annexin V PE Apoptosis Detection Kit (Cat number: 88-8102, eBioscience, USA) was used to label apoptotic cells according to manufacturer's instructions. LNCaP and DU145 cells with or without rCCL18 stimulation were seeded at a density of 5 × 10^5^ cells in 6-well plates. Annexin V-PE was added 48 hours later and apoptotic cells were identified with a flow cytometer according to manufacturer's protocol. Data were expressed as of three independent experiments.

### 2.9. Statistical Analysis

Thesoftware of SPSS version13.0 for Windows (SPSS Inc., IL, USA) and SAS 9.1 (SAS Institute, Cary, NC) was used for statistical analysis. Continuous variables were expressed as $\overset{-}{X}\pm s$.

## 3. Results

### 3.1. Upregulation of CCL18 at mRNA and Protein Levels in Human PCa Tissues

**Figure 1:**
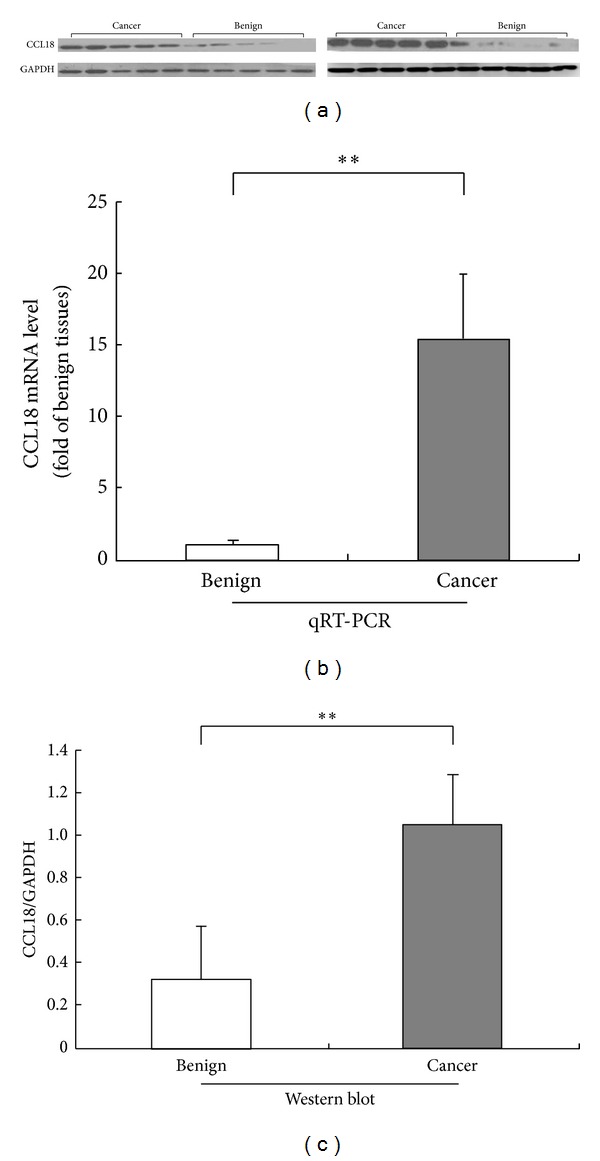
CCL18 mRNA and protein expression in prostate cancer (PCa) and adjacent benign prostate tissues detected by real-time quantitative RT-PCR assay (b) and western blot analysis ((a) and (c)). The statistical analysis showed that CCL18 mRNA average expression level (*n* = 10) was significantly upregulated in PCa tissues compared with those in noncancerous prostate tissues (*n* = 10, *P* < 0.001, (b)), which was consistent with the findings of western blot analysis on CCL18 protein expression (*n* = 10, PCa: 1.12 ± 0.24 versus benign: 0.26 ± 0.16, *P* = 0.017, (a) and (c)).

qRT-PCR and western blot analyses were performed to detect the mRNA and protein expression levels of CCL18 in 10 PCa tissues and 10 adjacent benign prostate tissues as shown in Figure 1. Relative expression ratio was defined as the expression levels of CCL18 mRNA to those of the internal reference gene, *β*-actin mRNA. The statistical analysis showed that CCL18 mRNA expression level was significantly upregulated in PCa tissues compared with those in noncancerous prostate tissues (*P* < 0.01, Figure 1(a)), which was consistent with the findings of western blot analysis on CCL18 protein expression (PCa: 1.06 ± 0.23 versus benign: 0.32 ± 0.24, *P* < 0.01, Figures 1(b) and 1(c)).

**Figure 2:**
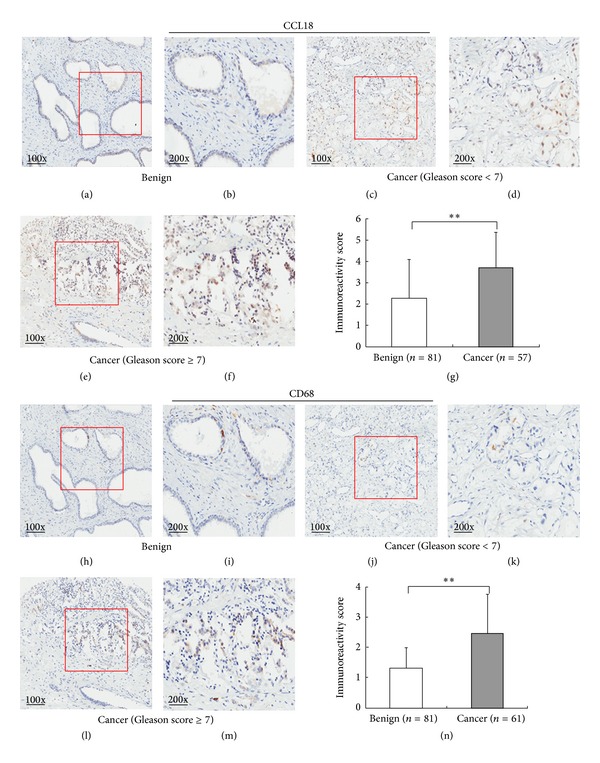
Expression pattern and localization of CCL18 protein in prostate cancer (PCa) and adjacent benign prostate tissues detected by immunohistochemistry analysis. ((a)–(f)) Immunostainings for CCL18 protein in PCa and adjacent benign prostate tissues. (g) Immunoreactivity score (IRS) of CCL18 protein in PCa tissues was higher than that in adjacent benign tissues (*P* < 0.001). ((h)–(m)) Immunostainings for CD68 protein in PCa and adjacent benign prostate tissues. (n) IRS of CD68 protein in PCa tissues was higher than that in adjacent benign tissues (*P* < 0.001).

In addition, the expression patterns and localizations of CCL18 in 80 PCa tissues and 95 adjacent benign prostate tissues were examined using immunohistochemical analysis. Please see detailed information on immunostainings of our TMA samples in supplementary files Supplementary Figure S1 and Table S1 in the Supplementary Material available online at http://dx.doi.org/10.1155/2014/230183. Representative pictures of immunostainings of CCL18 protein are shown in Figures 2(a)~2(f). In benign prostate tissues, CCL18 immunostainings occurred mainly in the cytoplasm of macrophages and the surrounding gland epithelial cells. In PCa tissues, positive immunostainings of CCL18 were observed in the cytoplasm of macrophages and cancer cells. In line with the results of western blot analysis, the IRS of CCL18 protein in PCa tissues was also significantly higher than those in benign prostate tissues (CCL18: PCa = 4.09 ± 1.81 versus benign = 2.31 ± 2.11, *P* < 0.01, Figure 2(g)). Since macrophage may secrete CCL18 protein which may be correlated with PCa, we also detected the expression of CD68, a marker for macrophage, in these tissue sections. As a result shown in Figures 2(h)~2(n), the expression of CD68 in PCa tissues was also higher than those in benign prostate tissues (CD68: PCa = 2.60 ± 1.30 versus benign = 1.26 ± 0.71, *P* < 0.01). More interestingly, the expression levels of CD68 in PCa tissues were significantly correlated with those of CCL18 (*R* = 0.337, *P* < 0.01).

### 3.2. Association of CCL18 Expression with the Clinicopathological Characteristics of PCa

The association of CCL18 expression with the clinicopathological features of PCa patients is shown in Table 1. In our cohort, the expression of CCL18 in PCa tissues was significantly correlated with Gleason score of patients (*P* = 0.034). The upregulation of CCL18 protein occurred more frequently in PCa tissues with high Gleason score of patients (GS ≥ 7).

### 3.3. CCL18 Promotes Tumor Invasion and Migration of PCa Cells

**Figure 3:**
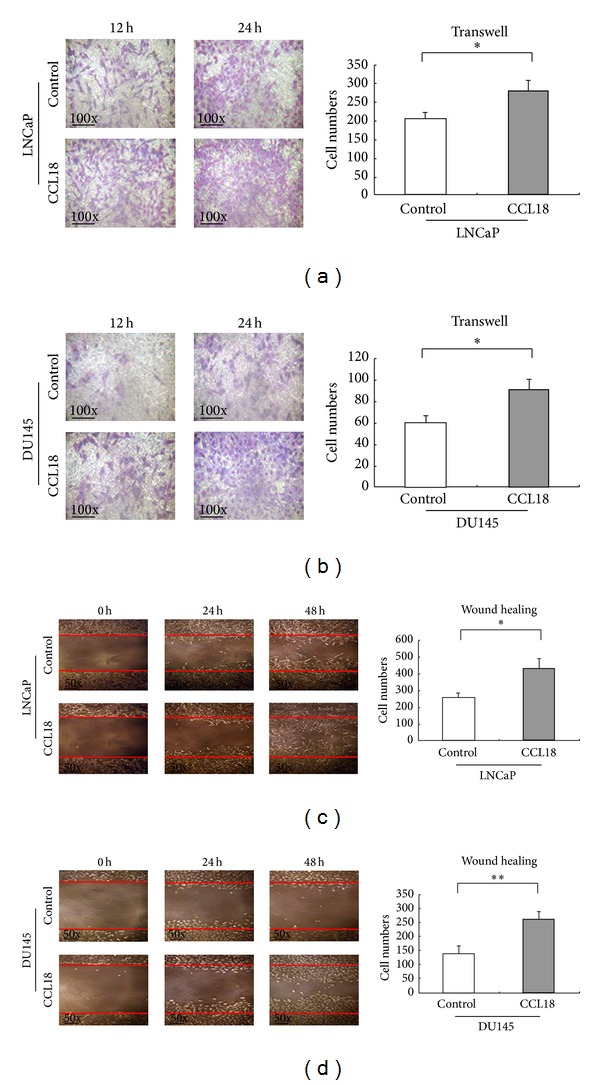
CCL18 promotes tumor invasion and migration of PCa cells. (a)-(b) Transwell analysis showing rCCL18 stimulation significantly enhanced the invasion activities of LNCaP (*P* = 0.015) and DU145 (*P* = 0.010) cells compared with those of control cells (evaluated at 100x magnification). (c)-(d) Wound-healing assays demonstrated that rCCL18 stimulation markedly increased the migration activities of LNCaP (*P* = 0.002) and DU145 (*P* < 0.001) cells (evaluated at 50x magnification).

Our data mentioned above suggested that the upregulation of CCL18 was significantly associated with malignant progression. Transwell assays clearly revealed that rCCL18 stimulation enhanced the invasion activities of LNCaP and DU145 cells compared with those of control cells (Figures 3(a) and 3(b)). In addition, wound-healing assays demonstrated that rCCL18 stimulation markedly increased the migration activities of LNCaP and DU145 cells (Figures 3(c) and 3(d)). These results indicated that CCL18 may improve the aggressive progression in PCa cell lines.

### 3.4. CCL18 Inhibits Apoptosis in PCa Cells

**Figure 4:**
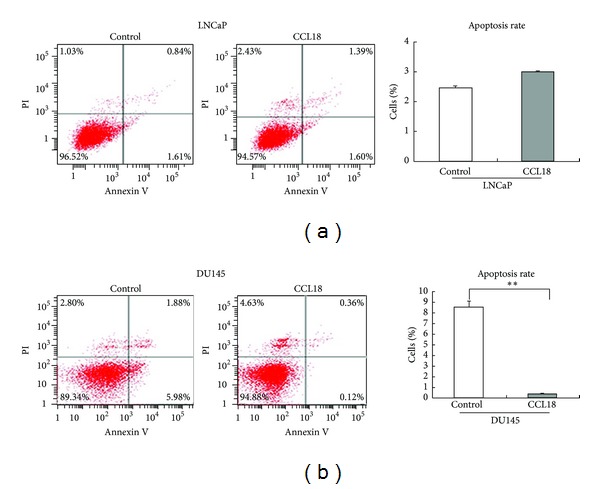
CCL18 inhibits apoptosis in DU145 cell line. Treated cancer cells with rCCL18 (20 ng/mL) 48 hours. (a) Apoptotic rate of rCCL18-simulated LNCaP cells was not significantly different. (b) However, rCCL18 treatment can significantly suppress the apoptosis of DU145 cells.

We next performed flow cytometric analysis to determine if cell apoptosis was affected by rCCL18. As shown in Figure 4, the apoptotic rate of rCCL18-simulated LNCaP cells was of no difference (Figure 4(a), 0.84 + 1.61 versus 1.39 + 1.60, *P* > 0.05). However, rCCL18 treatment can suppress the apoptosis of DU145 cells (Figure 4(b), 1.88 + 5.98 versus 0.36 + 0.12, *P* < 0.01). Upper results implied that CCL18 can prolong the survival time of cancer cells.

### 3.5. CCL18 Promotes Tumor Growth and Tumor Vascularization in RM-1 Homografts

**Figure 5:**
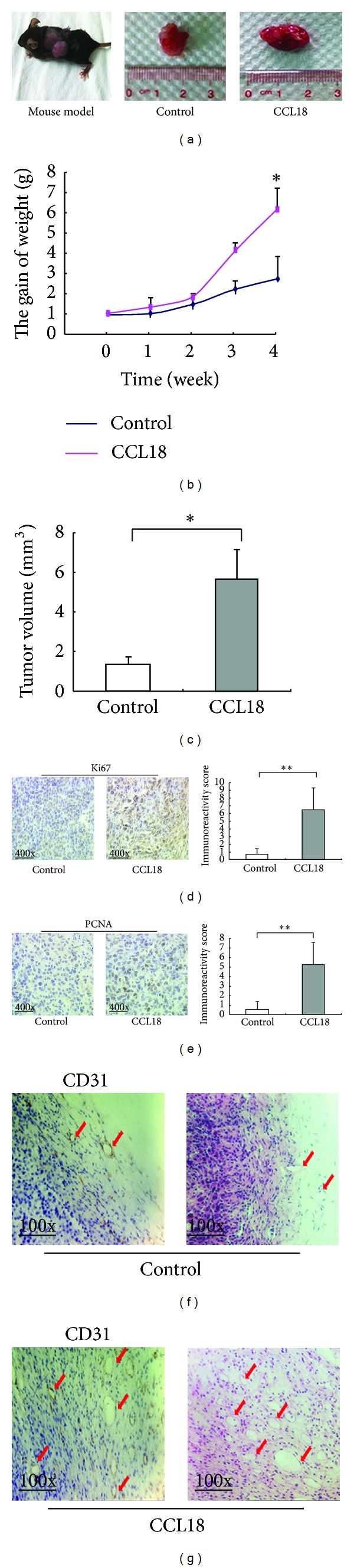
CCL18 treatment promotes tumor growth, expression pattern, and localization of Ki67, PCNA in RM-1 xenograft. Treat the mice with CCL18 for 4 weeks. (a) Tumor size. (b) The gain of weight. (g) Weight of each mouse was measured once a week (*P* = 0.04). (c) Tumor volumes were measured at 28th day and the results were used to plot the graphs (*P* = 0.032). In CCL18 treatment group, the expression levels of Ki67 ((d), *P* < 0.001) and PCNA ((e), *P* < 0.001) proteins were significantly higher than those in the control group. (f) CD31 staining and HE staining of control group. (g) CD31 staining and HE staining of CCL18 treatment group.

In order to validate the in vitro findings, we conducted in vivo experiments to test whether CCL18 could act as a tumor promoter in RM-1 homografts of mouse model. As the results show in Figure 5, rCCL18 stimulation significantly promoted tumor weight (*P* = 0.041, Figure 5(b)) and volume (*P* = 0.032, Figure 5(c)), which was consistent with its tumor promotive role in vitro.

Moreover, we detected the expression levels of cell proliferation markers PCNA and Ki67 in RM-1 homografts of mice model by immunohistochemistry analysis. As the results show in Figure 5, rCCL18 stimulation significantly enhanced the expression of Ki67 and PCNA proteins (both *P* < 0.01, Figures 5(d) and 5(e)).

In order to evaluate the influence of CCL18 on tumor vascularization, we detected the expression levels of the vascular endothelial marker CD31 in RM-1 homografts of mice model by immunohistochemistry analysis. As shown in Figure 5(g), rCCL18 stimulation significantly enhanced the expression of CD31 protein (*P* < 0.01).

## 4. Discussion

PCa represents a heterogeneous-multifocal disease with poor prognosis. The identification of novel and effective biomarkers may allow the development of new treatment strategies with less morbidity. It has been demonstrated that PCa may be an assemblage of distinct cell types that collectively construct a succession context referred to as tumor microenvironment, which is a complex mixture of cellular and noncellular factors, and plays an important role in tumor development and tumor cell dissemination [16]. The immune cells surrounding tumors and chemokine-crosstalk may affect tumor growth and progression [17]. Tumor associated macrophages (TAM), also known as tumor infiltrating macrophages, are one of the most important subgroup of immune cells in the tumor microenvironment [18]. Initially monocytes are recruited from the circulating blood to the tissue and differentiate in the two phenotypes M1- and M2-macrophages. The M1 phenotype is associated with proinflammatory pathways characterized by the release of proinflammatory cytokines and increased microbial killing [19]. The M2 phenotype shows a proangiogenic, prometastatic, and protumoral activity and is associated with tissue remodeling. TAMs are mostly M2-macrophages which secrete a specific pattern of chemokines [20]. Since CCL18 has been demonstrated to be released by TAMs which play important roles in malignant diseases, CCL18 has been increasingly considered to be involved in the pathogenesis and progress of various cancers. For example, Yuan et al. [21] found that the increased expression of CCL18 in colorectal cancer was an independent favorable prognostic factor in patients; Plönes et al. [22] revealed that serum level of CCL18 was increased in patients with non-small-cell lung cancer and predict a diminished survival time in adenocarcinomas; Wu et al. [11] also indicated that the preoperative serum level of CCL18 in gastric cancer patients was significantly higher than that of controls; Chen et al. [10] demonstrated that the serum level of CCL18 could clearly distinguish between women with breast cancers and healthy controls, and it was associated with worse prognosis; Urquidi et al. [13] also identified CCL18 as a highly accurate biomarker for bladder cancer detection. Shimura et al. [23] have demonstrated that TAMs may enhance the growth of PCa cells and release CCL18, which brought us to the hypothesis that CCL18 might function as a tumor promoter for PCa.

**Figure 6:**
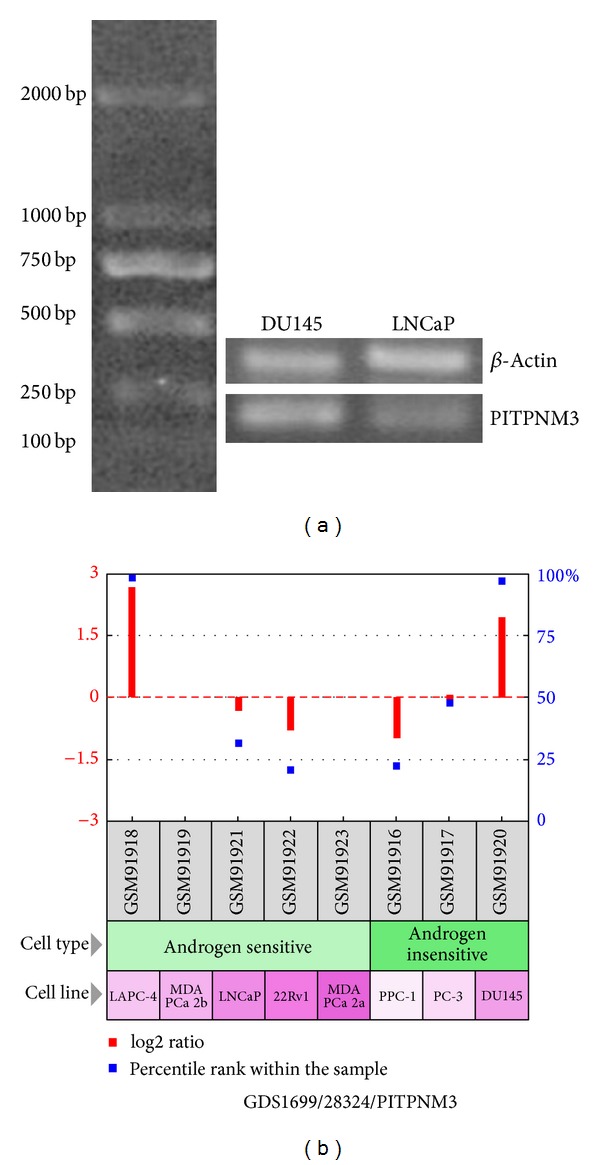
Expression level of PITPNM3 in DU145 cells was higher than that in LNCaP cells (a), which was in line with the data of GEO profiles (GDS1699/28324/PITPNM3, (b)).

We observed the mRNA and protein levels of CCL18 expression in PCa tissues were both higher than those in noncancerous prostate tissues. In our cohorts, CCL18 upregulation was associated with high Gleason score. Moreover, we found that rCCL18 stimulation in the in vitro system could significantly enhance the metastatic and the invasive potentials while reducing the apoptosis rate of DU145 but not LNCaP. In order to find out the reasons for this phenomenon, we detected the expression levels of CCL18 receptor PITPNM3 in LNCaP and DU145 cells. As a result, the expression level of PITPNM3 in DU145 cells was higher than that in LNCaP cells (Figure 6(a)), which was in line with the data of GEO profiles (GDS1699/28324/PITPNM3, Figure 6(b)). These data imply that DU145 may be more sensitive to CCL18 than LNCaP. Furthermore, the in vitro results were also supported by in vivo study using a subcutaneous homograft mouse model. We found that CCL18 could promote tumor growth in vivo by enhancing angiogenesis and expression of cell proliferation markers PCNA and Ki67.

In conclusion, our data offer the convincing evidence that the upregulation of CCL18 may be involved in the tumor aggressive progression of prostate cancer.

## Supplementary Material

Tissues Microarray Assay was bought from Shanghai Outdo Biotech Co, Ltd. which including 80 PCa tissues and 95 adjacent benign prostate tissues and the detailed clinical information (Age, Gleason score, Pathological Stage). In our result, CCL18 protein in PCa tissues was significantly higher than those in benign prostate tissues (CCL18: PCa= 4.09 ± 1.81 versus benign = 2.31 ±  2.11, P  <  0.01). The expression of CD68 in PCa tissues was also higher than those in benign prostate tissues (CD68 : PCa= 2.60 ±  1.30 versus benign = 1.26 ± 0.71, P < 0.01). the expression levels of CD68 in PCa tissues were correlated with those of CCL18 (R = 0.337, P < 0.01).

## Figures and Tables

**Table 1:** Association of CCL18 expression with the clinicopathological characteristics of PCa.

Clinical feature	IRS of CCL18 in our cohort		
	Case	$\bar{X}\pm s$	*P*-value
Age			
<66 years	15	2.67 ± 1.45	0.47
≥66 years	42	2.38 ± 1.25	
Gleason score			
<7	18	2.00 ± 0.91	0.034
≥7	38	2.68 ± 1.42	
Pathological stage			
II	45	2.42 ± 1.29	0.71
III	12	2.58 ± 1.38	
Metastasis			
No	57	2.46 ± 0.17	—
Yes	0	—	

Note: “—” means there are lack of relative information of patients in our cohort.
