# Supplementary material for: CC Chemokine Ligand 18 Correlates with Malignant Progression of Prostate Cancer
Source: Biomed Res Int. 2014 Aug 17;2014:230183. doi: 10.1155/2014/230183 (PMC4150478; doi:10.1155/2014/230183)
Supplement: Supplementary file 1 — Tissues Microarray Assay was bought from Shanghai Outdo Biotech Co, Ltd. which including 80 PCa tissues and 95 adjacent benign prostate tissues and the detailed clinical information (Age, Gleason score, Pathological Stage). In our result, CCL18 protein in PCa tissues was significantly higher than those in benign prostate tissues (CCL18: PCa= 4.09 ± 1.81 versus benign = 2.31 ± 2.11, P < 0.01). The expression of CD68 in PCa tissues was also higher than those in benign prostate tissues (CD68 : PCa= 2.60 ± 1.30 versus benign = 1.26 ± 0.71, P < 0.01). the expression levels of CD68 in PCa tissues were correlated with those of CCL18 (R = 0.337, P < 0.01). [file 230183.f1.pdf]

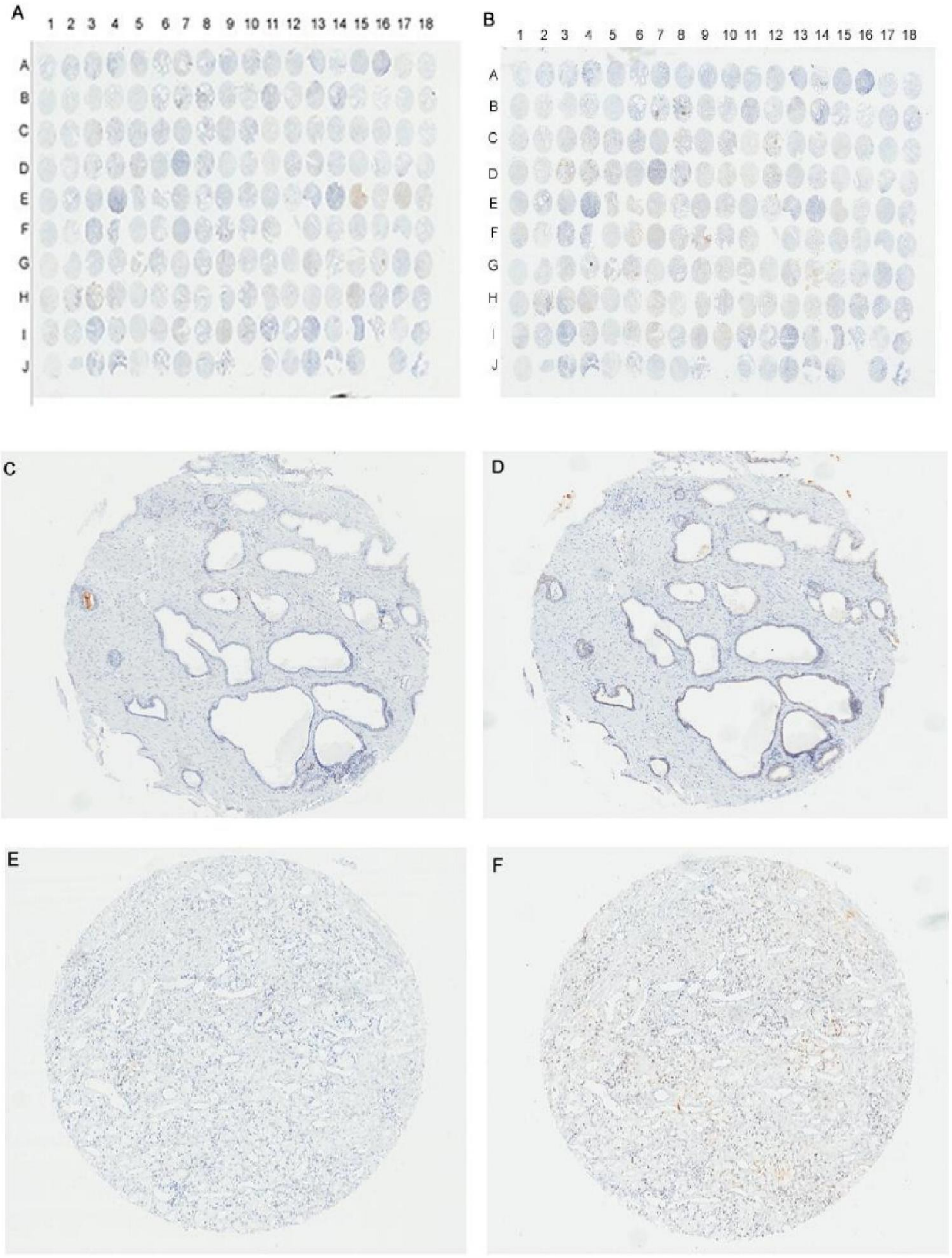
A
1 2 3 4 5 6 7 8 9 10 11 12 13 14 15 16 17 18
A
B
C
D
E
F
G
H
I
J
B
1 2 3 4 5 6 7 8 9 10 11 12 13 14 15 16 17 18
A
B
C
D
E
F
G
H
I
J
C
D
E
F

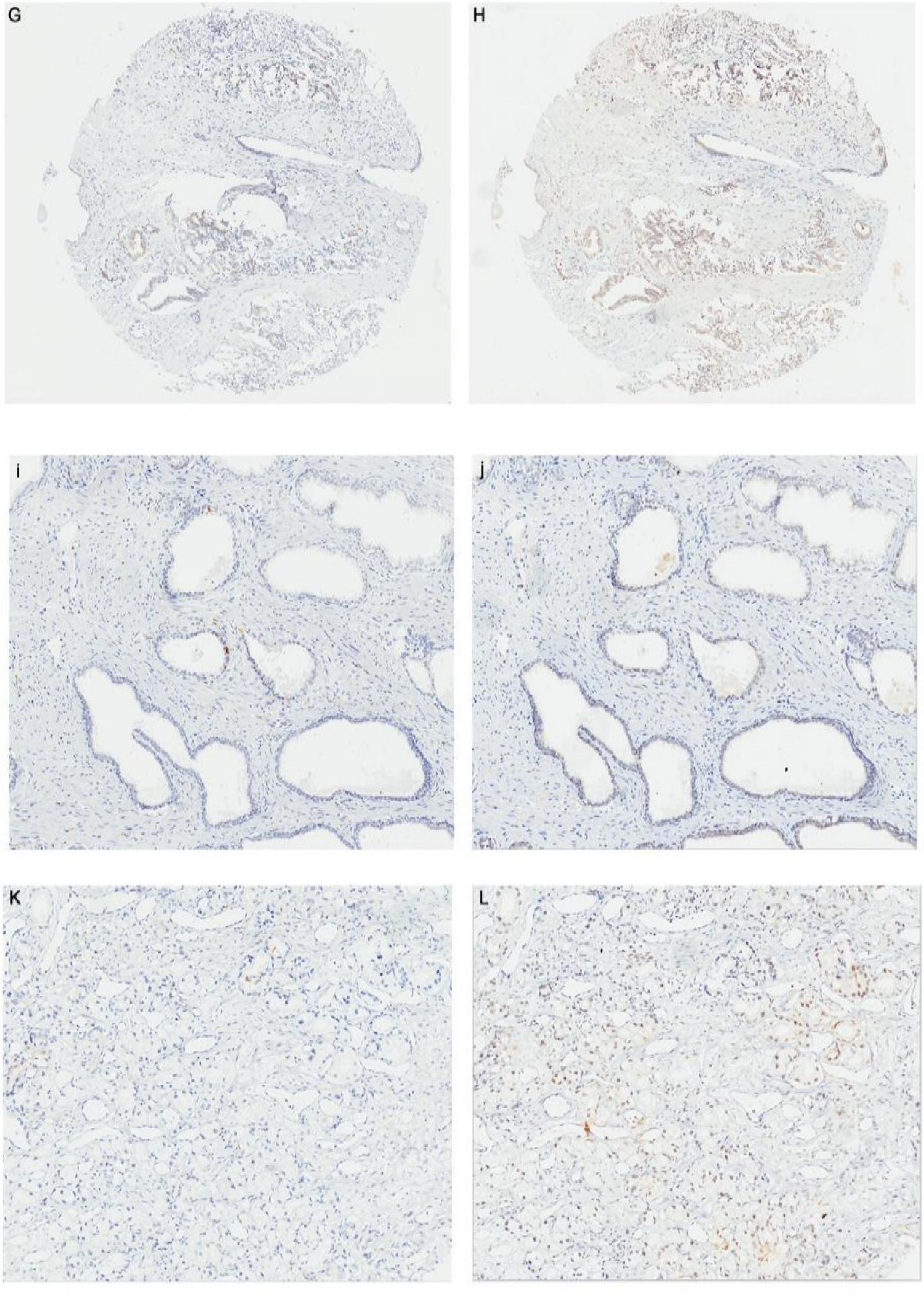
G
H
i
j
K
L

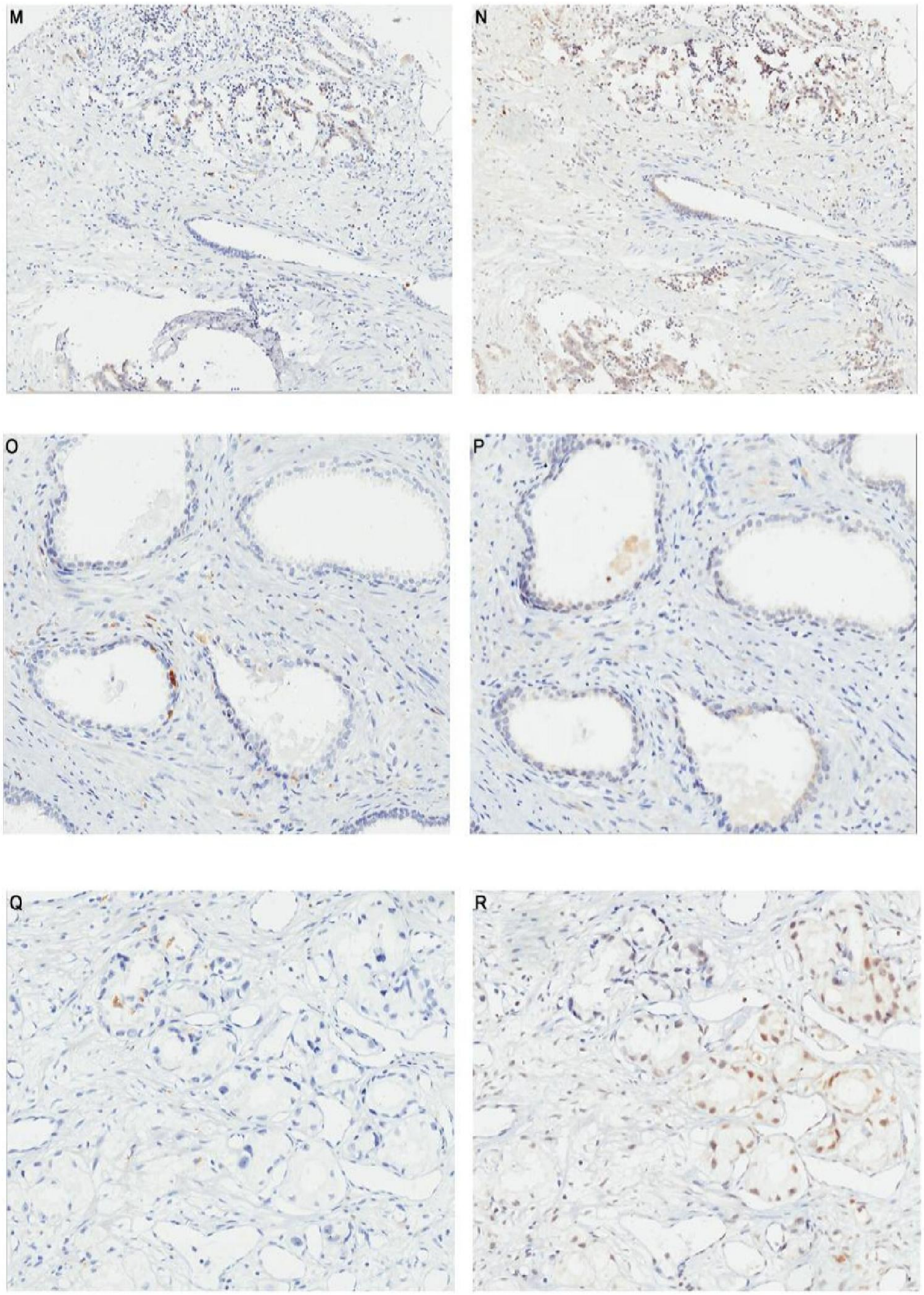
M
N
O
P
Q
R

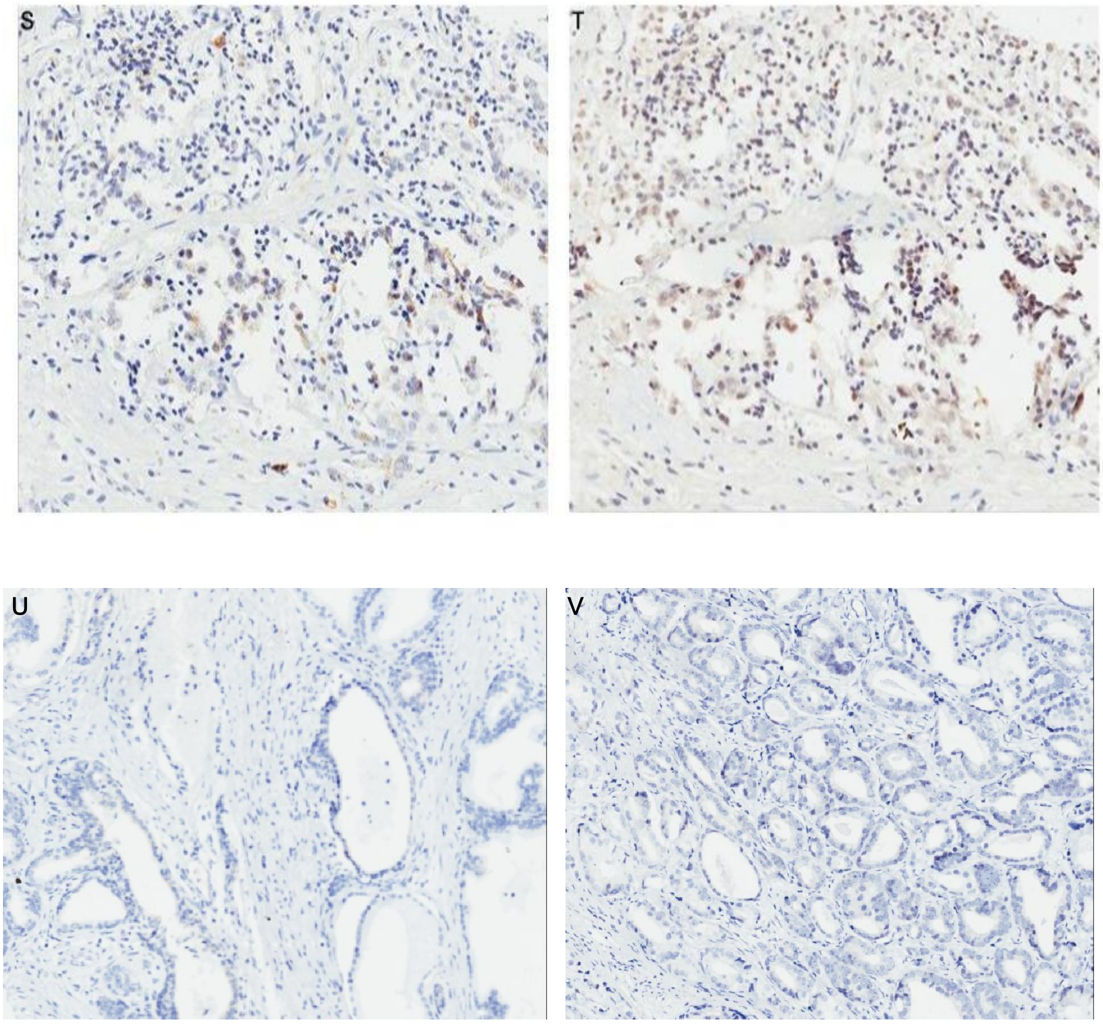

Supplementary Fig S1. Immunohistochemistry staining of our TMA Samples.

(A)CD68 immunostaining of TMA samples. (B)CCL18 immuostaining of TMA samples.

(C,E,G)CD68 Immunostaining of sample G8,F5,H7(Original magnification × 50),respectively.

(D,F,H)CCL18 Immunostaining of sample G8,F5,H7(Original magnification × 50),respectively.

(I,K,M)CD68 Immunostaining of sample G8,F5,H7(Original magnification × 100),respectively.

(J,L,N)CCL18 Immunostaining of sample G8,F5,H7(Original magnification × 100),respectively.

(O,Q,S)CD68 Immunostaining of sample G8,F5,H7(Original magnification×200),respectively. (P,R,T) CCL18 Immunostaining of sample G8,F5,H7(Original magnification×200),respectively.

(U,V)Negative controls, stained with isotype-matched control IgG
